# Mechanical behavior of sintered cross-shaped fivefold twinned Ag nanowires: Insights from a molecular dynamics study

**DOI:** 10.1007/s00339-026-09778-7

**Published:** 2026-06-10

**Authors:** Prabesh Ojha, Huadian Zhang, Manoj K. Shukla, Michael R Fiske, Jennifer E Edmunson, Shan Jiang

**Affiliations:** 1 Department of Mechanical Engineering, University of Mississippi, University, MS 38677 USA; 2 Environmental Laboratory, U.S. Army Engineer Research and Development Center, Vicksburg, MS 39180 USA; 3 Space Exploration Division, Amentum, NASA/Marshall Space Flight Center, Huntsville, AL 35812 USA; 4 ST23/Space Technology Development Branch, NASA/Marshall Space Flight Center, Huntsville, AL 35812 USA

**Keywords:** Molecular dynamics, Nanowire junction, Fivefold twins, Sintering, Quasi-icosahedral structure, Deformation mechanism

## Abstract

This study presents a molecular dynamics (MD) investigation of the sintering behavior and mechanical properties of cross-shaped silver (Ag) fivefold twinned nanowires (FTNWs). A model of two FTNWs arranged at 90° intersections was constructed and sintered at three temperatures (698, 898, 1098 K) with three heating rates (1.0, 0.1, 0.01 K/ps), followed by high-temperature relaxation and controlled cooling at 0.08 K/ps. After cooling, room-temperature relaxation was performed in the isothermal-isobaric ensemble using different time durations and reinitialized with different random seeds to ensure statistically independent velocity distributions and structural equilibration. Uniaxial tensile tests were then carried out at strain rates of 1.0 × 10^− 3^, 5.5 × 10^− 4^, and 1.0 × 10^− 4^ ps^− 1^. Effects of sintered temperature, heating rate, relaxation duration, and strain rate on yield strength were systematically analyzed. The results show that FTNWs sintered at lower temperature and higher heating rate tend to attain higher yield strength, attributed to limited atomic diffusion, which preserves the pre-existing fivefold twin boundaries (TBs) and structural integrity along the central axis. Additionally, higher strain rates yield greater tensile strength, while lower ones result in reduced strength. During deformation, FTNWs undergo nucleation and propagation of multiple stacking faults (SFs) that evolve into TBs and can eventually form a pair of quasi-icosahedral structures during necking and fracture; this mechanism consistently links to the formation of secondary fivefold twins around the original axis. For comparison, cross-shaped single-crystalline nanowires (SCNWs) were simulated under identical conditions: they exhibit ~ 18% lower yield strength but demonstrate markedly greater ductility (fracture strain is 4.5 times that of FTNWs), without showing any development of quasi-icosahedral structures during the tensile deformation.

**Supplementary Information:**

The online version contains supplementary material available at 10.1007/s00339-026-09778-7.

## Introduction

Metallic nanowires have attracted considerable attention due to their exceptional mechanical, thermal, and electrical properties, which arise from their high aspect ratios and nearly defect-free crystal structures [1]. These unique characteristics make them promising candidates for nanoelectromechanical systems, nanoelectronics [2, 3], and various sensing applications [4]. Unlike conventional bulk materials, where dislocation activity dominates deformation behavior, nanowires exhibit size-dependent mechanisms [5, 6], in which internal microstructural defects and surfaces play a crucial role in determining their mechanical response [7]. Among different metallic nanowires, those composed of face-centered cubic (FCC) metals frequently exhibit a distinctive fivefold twinning phenomenon [8]. This structure consists of five crystalline domains sharing a common axis in a pentagonal arrangement, which significantly enhances both mechanical strength and stability [9]. The twin boundaries (TBs) formed in twinned Ag nanowires act as effective barriers to dislocation motion, contributing to increased strength under applied mechanical loads [10]. In the past, many studies have reported their presence across a variety of nanostructures, including nanoparticles [10], nanoclusters [11, 12], nanowires [13], and nanorods [14]. Understanding how these TBs influence the deformation behavior of nanostructure-based structures is essential for ensuring their reliability [15, 16]. To explore these complex atomic-scale deformation mechanisms, atomistic simulations offer a powerful tool; for example, molecular dynamics (MD) enables better simulation and predictions of formation [17], and interaction [18] of twin boundary (TB), surface energy anisotropy [19], and sintering dynamics [20]. Previous MD studies, particularly those performed with embedded atom method (EAM) potentials [21], have shown that during sintering, parameters such as heating rate [22, 23] and temperature [24] can significantly affect neck formation [25, 26], grain boundary evolution [27], and the resulting mechanical properties of the nanostructures [7].

While the mechanical behavior of the above-mentioned fivefold twinned nanowires (FTNWs) has been extensively investigated [9, 28, 29], limited attention has been given to the sintered FTNWs, particularly regarding how fast-laser sintering influences their structural and mechanical stability. Such knowledge is currently lacking but is required for improved design of portable and flexible electronics using networks of sintered nanowires [30]. Previous studies have shown significant size-dependent behavior in FTNWs [31]. The present simulations were performed for a specific cross-shaped FTNW with a fixed wire diameter of 7.2 nm. Therefore, the observed strength and deformation trends should be interpreted in the context of this model. Additionally, the successful laboratory preparation of twinned nanowires has revealed several novel features, including an enhanced yield strength in penta-twinned Ag nanowires [32], an increased elastic modulus with Young’s modulus increasing [33], and a unique coiling motion [34] induced by the intrinsic fivefold axis. Research on Ag nanowires with fivefold twin structures has demonstrated that TBs restrict dislocation motion as the diameter decreases, leading to size-dependent plasticity and increased strength [35]. MD studies show that under combined loading conditions, especially tension after pre-torsion [36], fivefold deformation twins (FDTs) tend to nucleate in the necking region of the single-crystalline nanowires (SCNWs), while under continued uniaxial tensile loading, they can reorganize into a stable quasi-icosahedral structure composed of multiple conjoint twins in the necking region of FTNWs [37]. In this work, we (i) systematically studied the interplay of sintering and tensile loading in a FTNW network under varying conditions, (ii) discovered a unique quasi-icosahedral structure formed during the deformation, and (iii) compared the mechanical behavior of sintered cross-shaped SCNWs and FTNWs under tensile loading. These atomistic investigations aim to clarify the interaction between sintering processes and mechanical loading, providing detailed insight into the underlying deformation pathways of FTNWs.

Understanding the complete mechanical response of nanowires is crucial for device applications under combined mechanical and thermal stresses, as mechanical deformation affects electronic properties like band structure and carrier mobility. Although techniques like atomic force microscopy and in-situ nanomechanical testing have advanced [38–41], capturing transient atomic-scale events remains challenging due to spatial and temporal resolution limits. Atomistic simulations help bridge these gaps by providing time-resolved insights into stress, surface effects, and twinning dynamics in nanowire networks under complex loading conditions. This study offers predictive frameworks for optimizing the mechanical reliability of FTNWs in high-strain, high-temperature environments. Despite advances, research on the sintering behavior of FTNWs at various heating rates is limited. This work fills this gap, providing a theoretical foundation for future studies on the thermal and mechanical behavior of FTNW junctions in nanoscale networks, which are key to the additive manufacturing of flexible electronics. Although this study offers atomic-scale insights into the sintering and failure of crossed FTNWs, it remains idealized, excluding substrate support and adhesion, which experimental work has shown to influence fracture and deformation [42]. Therefore, the present results should be viewed as atomistic trends for an unsupported FTNW model, rather than direct predictions for supported nanowire devices.

Moreover, the outcomes of the present atomistic research will be beneficial for the development of larger-scale, coarse-grained MD models that capture both the individual and collective behavior of nanowire networks. Integrating these simulations with mesoscale and continuum models will enhance understanding of deformation mechanisms and network integrity. In the future, machine learning models trained on atomistic datasets will enable rapid evaluation of structure-property relationships, advancing the design and optimization of high-performance nanowire-based networks.

## Simulation method

### Atomic force field

The present work employs MD simulations to investigate the mechanical and structural behavior of the cross-shaped Ag FTNWs at different sintered temperatures and heating rates. The atomic interactions among Ag atoms are characterized by the EAM potential [21]. This method provides an excellent representation of transition metals exhibiting an FCC structure. The EAM force field handles atomic interactions during simulation. The total energy present in the system is calculated using the following,

1 $$\:{E}_{total}=\sum\:_{i}{F}_{i}\left({\rho\:}_{i}\right)$$

where $\:{F}_{i}$ represents the embedding energy, $\:{\rho\:}_{i}$ is the background electron density at the position $\:{R}_{i}$ without the contribution of the atom *\:i*. This method assumes that each atom in the system interacts with the locally uniform electron ‘gas.’ This embedding energy $\:{F}_{i}$ is defined as the energy of the atom inside the uniform electron ‘gas’ relative to its energy when separated from the gas. The electron density function represents the energy per atom at the impurity location, plus an electrostatic interaction. Therefore, the total energy is the sum of all individuals and is defined as,

2 $$\:{E}_{total}=\sum\:_{i}{F}_{i}\left({\rho\:}_{i}\left({R}_{i}\right)\right)+\frac{1}{2}\sum\:_{i,j(=1)}\varphi\:\left({R}_{ij}\right)$$

where $\:\varphi\:$ is defined as a short-range electrostatic pair potential, and $\:{R}_{ij}$ is the distance between atoms *i* and *j*.

### Modeling and equilibration of samples

**Fig. 1:**
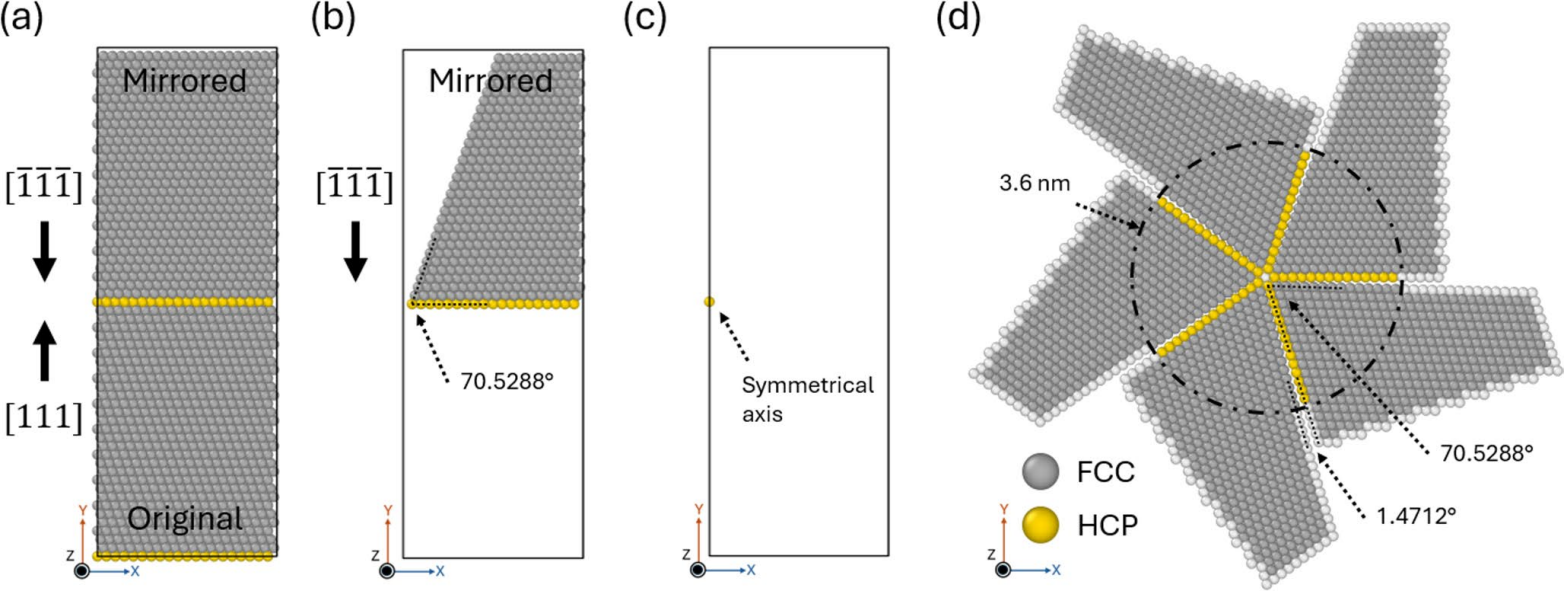
The construction of a base FTNW: (**a**) the (111) TB model, (**b**) the cut geometry as one part of the base FTNW, (**c**) the extracted axial atoms serving as the symmetrical axis of the base FTNW, and (**d**) the base cylindrical FTNW assembly with a radius of 3.6 nm

**Fig. 2:**
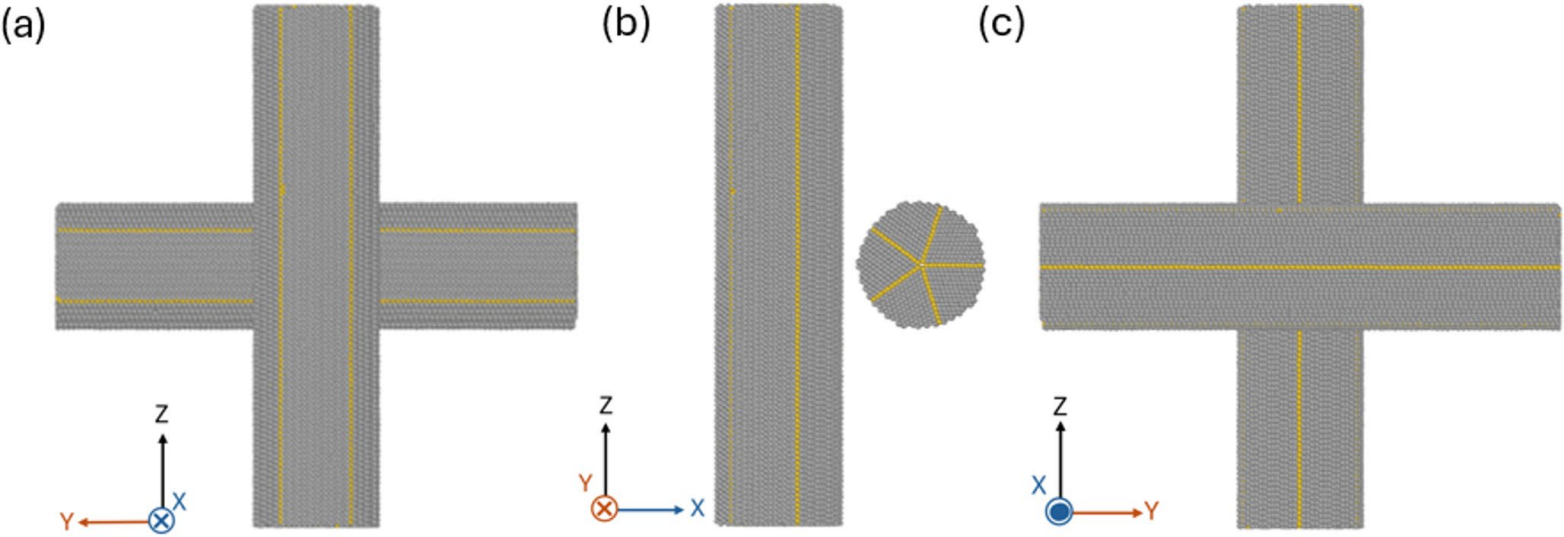
The initial cross-shaped FTNW model in different orientations (**a**) left view, (**b**) front view, and (**c**) right view

The base fivefold twin model (including 70,600 atoms) was constructed using open-source tools ATOMSK [43] and OVITO [44], featuring TBs oriented perpendicular to the longitudinal axis and exhibiting symmetry along the same axis, with a 72° angle between adjacent boundaries. A TB is characterized as a mirror plane between two crystals. Therefore, constructing such a boundary with ATOMSK is straightforward: first, create a crystal with the desired orientation; then, generate its mirror image to obtain the second crystal; and finally, stack the two crystals together. In this study, a $\:\left(111\right)$ TB was designated for building the base fivefold Ag model. Firstly, the Ag crystal unit cell was established with the orientation of $\left[11\stackrel{-}{2}\right]$, $\:\left[111\right]$, and $\:\left[\stackrel{-}{1}10\right]$ along the *x*, *y*, and *z* axes, respectively. The unit cell was then replicated 10 times in all directions to form a supercell. The final $\:\left(111\right)$ TB Ag model was created by stacking the original supercell and the mirrored one with respect to that plane, as illustrated in Fig. 1(a). Preserving the crystal structure attributes, i.e., FCC and hexagonal close-packed (HCP) structures, in the simulation box with periodic boundary conditions, an angle of 70.5288° shaped geometry was cut from the TB model, and the axial atoms serving as the center of FTNW were extracted (see Figs. 1(b) and 1(c), respectively) using OVITO. Consequently, as illustrated in Fig. 1(d), the base fivefold twin model can be assembled through the precise replication and rotation (72°) of the cut geometry. It is observed that there is a 1.4712° gap between every twofold, indicating a limitation in constructing nanowires with larger radii and the need for geometry optimization. Hence, a cylindrical base fivefold twin model was subsequently extracted, with a radius of 3.6 nm (circled in Fig. 1(d)). Then the cylindrical sample was replicated longitudinally in the *z*-direction, forming the FTNW with a diameter of 7.2 nm and a length of about 29.0 nm. The two FTNWs were positioned surface-to-surface in a cross arrangement at their central location, as shown in Fig. 2. For all the figures, including atomic configurations visualized hereinafter using Common Neighbor Analysis (CNA), FCC atoms are shown in gray, those in HCP in gold, and the disordered or surface atoms in white, allowing direct identification of defects and structural transformations during deformation. The TBs appear as single atomic layers of HCP atoms sandwiched between FCC atoms.

In perfect FCC structures, atomic layers are arranged in a sequential pattern. If the planar ordering is slightly disrupted and the sequence is disrupted by the addition or removal of atoms, stacking faults (SFs) are formed. SFs relate to errors in the perfect stacking sequences. Intrinsic SFs (ISF) and extrinsic SFs (ESF) may be viewed as simple variations from this ideal stacking sequence. For example, ISF is formed when a pair of atomic planes is removed from the ideal stacking sequence, while the ESF is formed by inserting a pair of planes. These SFs subsequently form the TBs. Consequently, the TBs can be visualized as continuous single-layer gold planes dividing the FCC domains. Such a visualization method can effectively capture the evolution of SFs, twin formation, and quasi-icosahedral structures within the nanowire during tensile deformation.

The simulation setup started with the minimization of an initially generated FTNW, which stabilized the nanowire’s atomic structure by removing residual stresses using the conjugate gradient method. The simulation tracked the system’s potential energy during the minimization and ensured that a relatively stable atomic configuration was reached before thermal equilibration. In the next step, the system was equilibrated at room temperature (298 K) using a Nosé-Hoover thermostat and barostat, under periodic boundary conditions along the longitudinal direction. The thermostat maintained the system temperature at 298 K throughout the equilibration process. Also, the pressure was maintained at 0 bar by using the barostat. The timestep was set to 2 fs throughout the simulation. When the single nanowire was well thermally relaxed, it was replicated and rotated by 90°. The original and the rotated FTNWs were then combined to form a cross-shaped model with an initial gap of 0.5 nm, as shown in Fig. 2. The cross-shaped model of FTNWs was then equilibrated using the same NPT ensemble at 298 K, simulating the solid-state sintering process, which resembles metallic powder fusion in an ambient environment.

### Sintering simulation process

**Fig. 3:**
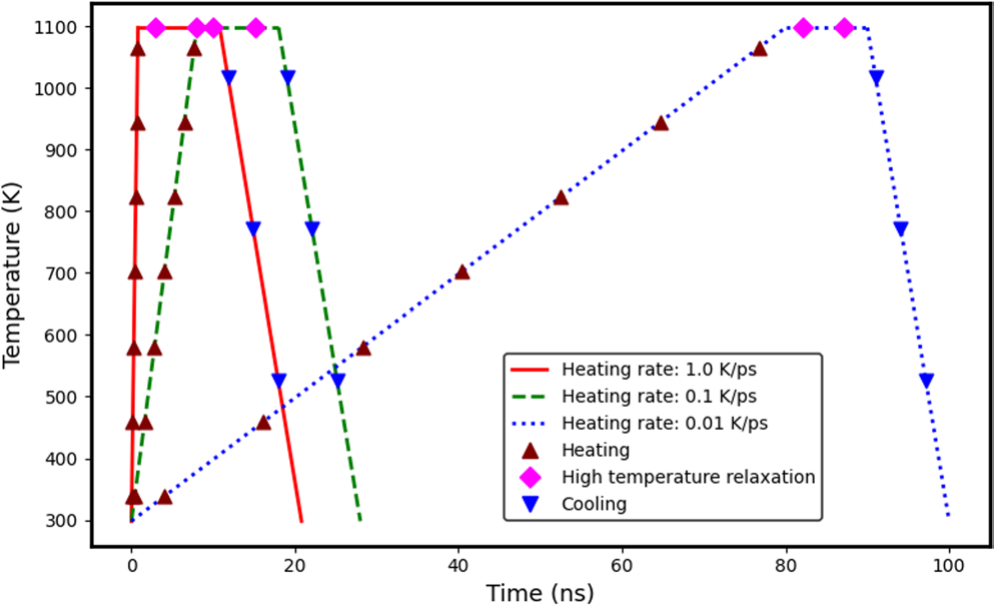
Heating, high-temperature relaxation, and cooling phases in the sintering process for a final temperature at 1098 K of the cross-shaped FTNWs at different heating rates of 1.0, 0.1, and 0.01 K/ps

A non-periodic boundary condition was used in the *x*-direction. At the same time, we applied periodic boundary conditions along the other two *y-* and *z-*directions to mimic an infinitely long nanowire. After conducting NPT for 5 ns at 298 K, the system’s temperature was linearly ramped up to model the laser sintering process, roughly replicating the temperature rise reported during selective laser sintering (SLS) of metal powders [45–47]. Although SLS involves complex effects such as oxidation, ambient atmosphere, and laser–matter interactions, the present study employs an idealized thermal process to isolate the intrinsic atomistic mechanisms of cross-shaped FTNWs. While experimental studies indicate that heating path and oxide-related processes influence nanowire evolution [48], the simplified framework used here enables a clear understanding of fundamental structure–property relationships. The system was heated at rates of 1.0, 0.1, and 0.01 K/ps to final temperatures of 698, 898, and 1098 K, all below the melting point of Ag, as shown in Fig. 3. These heating rates were chosen to differ by an order of magnitude (10 times) from one another, which helps provide a detailed analysis of the dynamic behavior of FTNWs across a wide range of thermal conditions. After heating, a high-temperature relaxation was performed for 10 ns in the NPT ensemble to structurally equilibrate the rapidly heated nanowire. This relaxation procedure helps to achieve a stable joint between the NWs at the intersection point. Following relaxation, a follow-up solidification process was performed using the NPT ensemble. In this process, the NWs are gradually cooled to 298 K at a rate of 0.08 K/ps, which approximately replicates the SLS solidification process under ambient conditions after the laser tip is removed. During the heating and cooling phases, the thermodynamic properties are recorded every 100 timesteps. Finally, an additional room-temperature relaxation was performed for different durations to eliminate any residual stresses in the NW model caused by dynamic cooling. During the room-temperature relaxation stage, time durations of 2, 4, 6, 8, 10, 12, and 14 ns, along with 10 different random seeds for each time duration of 1 ns, were considered to obtain better averaged ultimate tensile strength (UTS) by minimizing statistical fluctuations arising from atomic configurations.

Subsequently, the thermodynamic properties and atomic structure during sintering are visualized and investigated using the CNA method, which serves as an effective filtering approach for classifying atoms in crystalline systems by characterizing their local structures [49]. The key thermodynamic properties collected are the mean square displacement (*\:MSD*) and radius of gyration ($\:{R}_{g}$), which are used to characterize the diffusive behavior. The *\:MSD* measures the sum of the average distance traveled by each atom during the sintering process, which is given as,

3 $$\:MSD=\frac{1}{N}\sum\:_{i=1}^{N}{\left[{r}_{i}\left(t\right)-{r}_{i}\left(0\right)\right]}^{2}$$

where *\:N* is the total number of atoms in the system, *\:t* is the time, and *\:r* is each atom’s current position. The *\:MSD* is calculated using the atom’s coordinates and position at the beginning of the heat-sintering process. The $\:{R}_{g}\:$is also calculated as another essential component of the sintering process, which better represents the size change in the system. It is the root of the mean square distance between atoms in a group as determined by their center of mass and expressed as,

4 $$\:{R}_{g}=\sqrt{\frac{1}{M}\sum\:_{i=1}^{N}{{m}_{i}\left({r}_{i}-{r}_{cm}\right)}^{2}}$$

where *\:M* is the mass of the atoms, *\:r* is each atom’s current position, $\:{r}_{cm}$ is the center of mass position, and the subscript *\:i* runs over all the atoms present in the system.

### Tensile deformation process

The final sintered cross-shaped model of FTNWs underwent uniaxial tension along the *z*-direction at a different strain rate ($\:\dot{\epsilon\:}={\Delta\:}{\epsilon\:}_{zz}/{\Delta\:}t$). Metallic materials are widely recognized as strain rate sensitive, as demonstrated by both experimental and computational research. Under tensile loading, the applied strain rate has a significant impact on the mechanical behavior of FTNWs. MD simulations have also shown that higher strain rates increase yield strength and alter deformation mechanisms in FTNWs [9]. To understand the mechanical response of FTNWs under varied loading conditions, a study of their strain rate sensitivities is essential. In this study, the final sintered model of FTNWs is subjected to three strain rates: 1.0 × 10^− 3^, 5.5 × 10^− 4^, and 1.0 × 10^− 4^ ps^− 1^.

To examine the mechanical behavior of materials under deformation, precise control of strain rate is essential in MD simulations. The *fix deform* command adjusts the simulation box in the NVE ensemble, which represents an isolated system in which the number of particles (N), volume (V), and total energy (E) remain constant throughout the simulation. This *fix* uses the velocity formulation of the Stoermer-Verlet time integration scheme, also known as velocity-Verlet, which updates the positions and velocities at each timestep [50]. This technique is enabled by the LAMMPS algorithm, which remaps atom positions and velocities without introducing volumetric effects, particularly when periodic boundary conditions are applied in the *z*-direction. By distributing the applied strain evenly throughout the nanowire, this method produces a realistic simulation of its mechanical response at various strain rates. An essential parameter in the simulation of tensile loading of FTNWs is the normal strain increment along the nanowire at each time step, represented by $\:{\Delta\:}{\epsilon\:}_{zz}$. To determine the deformation, the change in the simulation box’s length along the *z*-direction is equivalent to the change in the nanowire’s length. The virial stress formula [51] was used to calculate the atomic stress, and the stress tensor components were determined based on the per-atom stresses and derived statistically using the virial theorem of Clausius [52, 53], which is given as,

5 $$\begin{array}{c}{\sigma\:}_{ij}^{V}=\frac{1}{V}\sum\:_{\alpha\:}\\\left[\frac{1}{2}\sum\:_{\beta\:=1}^{N}\left({R}_{i}^{\alpha\:}-{R}_{i}^{\beta\:}\right){F}_{j}^{\alpha\:\beta\:}-{m}^{\alpha\:}{v}_{i}^{\alpha\:}{v}_{j}^{\alpha\:}\right]\end{array}$$

where *\:i* and *\:j* represent the values in the *x*-, *y*-, and *z*-direction, while $\:\beta\:$ denotes the values from 1 to *\:N* neighboring atoms of $\:\alpha\:$. Here $\:{R}_{i}^{\alpha\:}$ is the position of the atom $\:\alpha\:$ along the direction of *\:i*, $\:{F}_{j}^{\alpha\:\beta\:}$is the force that is extended along the direction, *\:V* denotes the volume, $\:{m}^{\alpha\:}$ denotes the mass of an atom $\:\alpha\:$, and $\:{v}^{\alpha\:}$ is its thermal excitation velocity. Later, the stress-strain curves were obtained through statistical analysis of the simulation results. The stress values were computed as volumetric averages of all per-atom stress tensor components, while the strain values were determined from changes in the simulation box length. Both stress and strain were recorded at 200-timestep intervals. In addition to determining the mechanical behavior of the sintered FTNWs, local atomic structures visualized by using CNA were also identified to investigate the deformation mechanisms.

## Results and discussions

### Solid-state sintering at room temperature

**Fig. 4:**
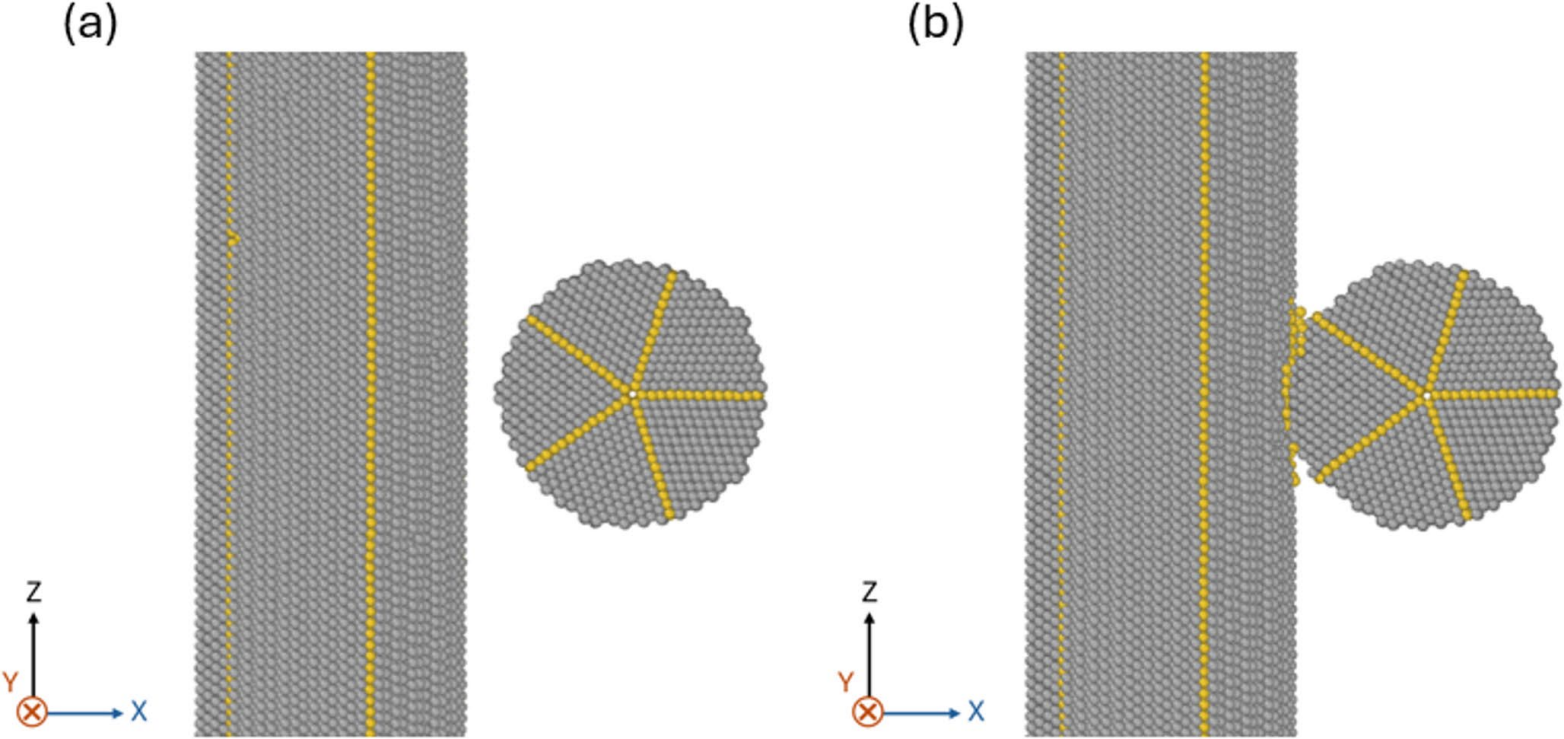
Configurations of the cross-shaped FTNWs (**a**) before and (**b**) after a solid-state sintering process at 298 K. The two nanowires, initially separated by 0.5 nm, form a neck connection at the point of contact over time. Disordered and surface atoms are removed for better visualization of the neck connection

The phenomenon of solid-state sintering can occur for all cross-shaped nanowires without any laser irradiation during the thermally equilibrated process at room temperature, due to the strong interatomic forces on the surfaces of the FTNWs. This behavior arises from the ultrafine size of atoms and a significantly considerable atomic potential [54]. Figure 4 illustrates the progression of nanowire deformation during room-temperature solid-state sintering, as captured in snapshots taken before and after the process. Initially, the two nanowires are separated by 0.5 nm with no laser heating, and the system temperature is 298 K, as shown in Fig. 4(a). As the equilibrium time increases, the nanowires move closer, eventually connecting and forming a neck at the point of contact, as shown in Fig. 4(b). After sintering, as shown in Fig. 4(b), a clear neck forms at the point of contact between the two nanowires, with atoms migrating from the free surface to the contact interface mediated by planar defects, including many HCP atoms. The number of HCP atoms increases from 12,870 to 13,397, corresponding to about a 4.1% rise, indicating a noticeable atom rearrangement from FCC to HCP in the neck junction region. This 298 K solid-state sintered model is used for a sequence of laser heating simulations, as discussed in the next section.

### $\:{M}{S}{D}$ and ${R}_{g}$

**Fig. 5:**
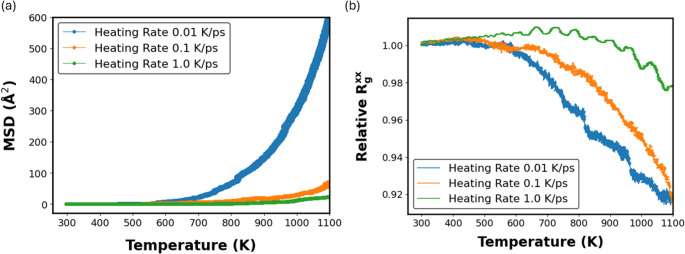
Temperature dependence of (**a**) *\:MSD* and (**b**) relative $\:{R}_{g}^{xx}$ during the sintering of a cross-shaped FTNW structure at varied heating rates

This section presents the utilization of *\:MSD* and relative $\:{R}_{g}$ to analyze the atomic behavior and to study the sintering mechanism during the laser heat sintering process. This *\:MSD* calculation measures the average distance traveled by atoms in the system and effectively approximates their diffusion. The data are obtained from simulations at three heating rates (1.0, 0.1, and 0.01 K/ps). Figure 5(a) shows the graphical representation of *\:MSD* vs. temperature curves obtained for these rates. The *\:MSD* shows the diffusion of atoms and captures the growth of the neck connection at the nanowire intersection, driven by the sudden increase in the *\:MSD* gradient. The *\:MSD* curve shows that across all heating rates, the *\:MSD* value remains at a minimal level below 600 K, corresponding to the solid-state of nanowires, indicating that the cross-shaped model of nanowires remains stable with minimal atomic movement. At temperatures below 600 K, atomic vibrations are confined to the equilibrium position, and thermal energy is insufficient to cause significant atomic diffusion. As the temperature rises from 600 to 800 K, the *\:MSD* increases gradually, indicating that the nanowire has reached its thermal softening point, which in turn leads to a slight increase in atomic mobility. At higher temperatures above 800 K, a sharper rise in *\:MSD* is observed, especially for nanowires heated at a slower heating rate, indicating that atoms diffuse more readily from their original state and vibrate more energetically, with greater mobility. The extent of disordered atoms also becomes maximum at higher temperatures. Specifically, as the temperature rises above 600 K, the *\:MSD* at the lowest heating rate of 0.01 K/ps shows a more pronounced rise than at higher heating rates. This trend, in which the steepest *\:MSD* increase occurs at the slowest heating rate, around 700 K, aligns with previous MD research on neck growth during laser sintering of solid Ag nanoparticles, with the consideration of the heating rate effect [54].

The characteristics of structural evolution during the heating process were examined through the calculation of the $\:{R}_{g}$. This quantity provides a direct measure of the distribution of atoms within the nanowire, reflecting the extent of its structural change as the temperature increases. The gyration tensor, $\:{R}_{g}$, was calculated from the atomic coordinates to quantify the mass distribution related to the instantaneous center of mass. The diagonal components, particularly with respect to the *x*-direction, i.e., *xx*-component of the relative $\:{R}_{g}$ tensor ($\:{R}_{g}^{xx})\:$with respect to temperature is shown in Fig. 5(b). To calculate the relative $\:{R}_{g}^{xx}$, a normalized measure is defined as the ratio of the instantaneous to the initial gyration components in the *x*-direction. Therefore, it provides a straightforward measurement of the coalescence extent during the heat sintering process.

The relative $\:{R}_{g}^{xx}$ shows a decrease with increasing temperature, as seen in Fig. 5(b), due to structural changes to a more “close-packed” state in the system as it is heated. The rate of decrease is more pronounced at a slower heating rate, as it has sufficient time to equilibrate and undergo such structural changes. This slow-heating-rate behavior enables a controlled sintering process, resulting in a more stable final structure. In contrast, systems heated more rapidly maintain higher relative $\:{R}_{g}^{xx}$ values across the temperature range, indicating limited time for equilibration and structural reorganization.

**Fig. 6:**
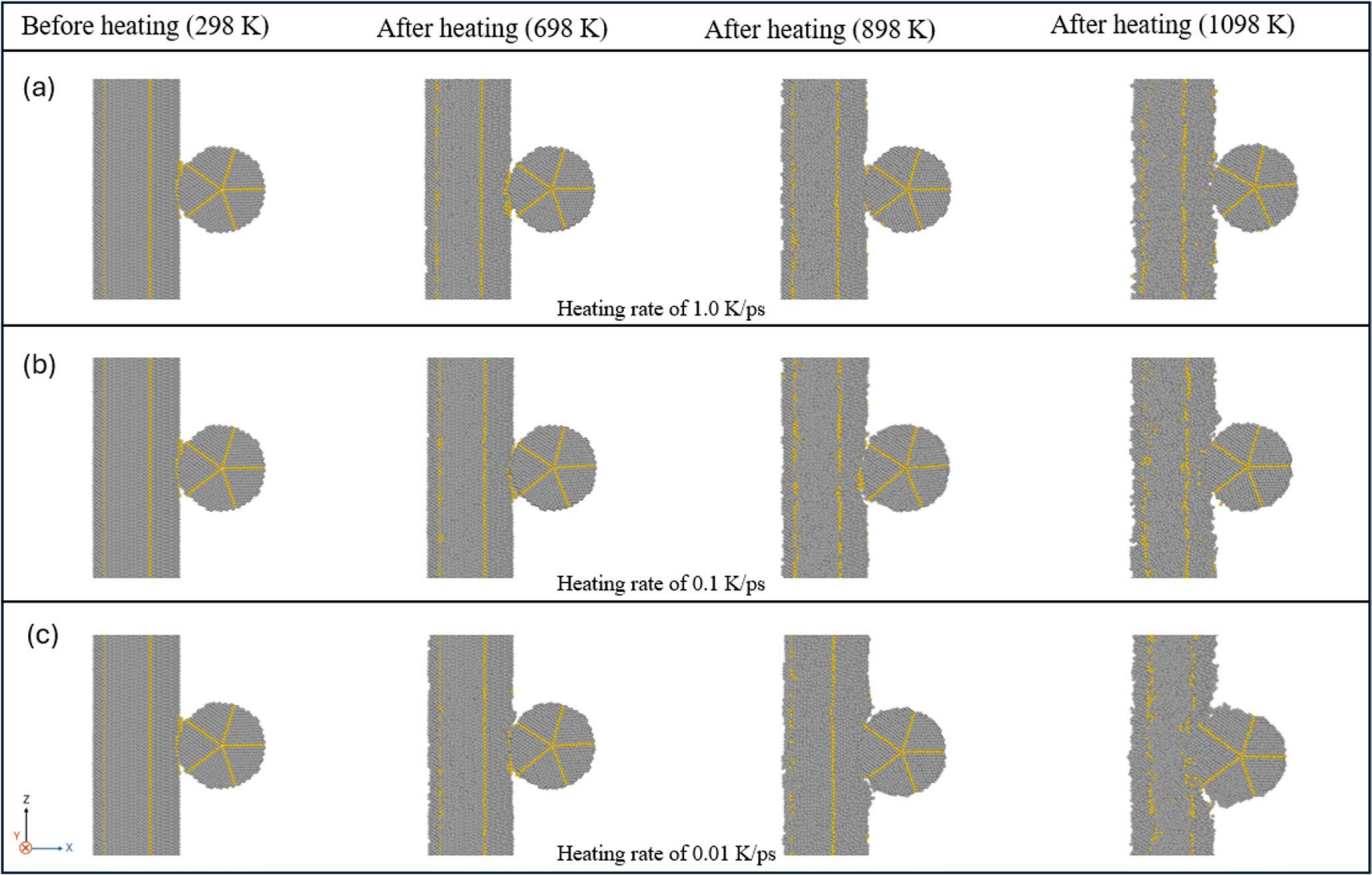
Snapshots of the sintered FTNWs before heating at 298 K and heated to 698, 898, and 1098 K at a heating rate of (**a**) 1.0, (b) 0.1, and (**c**) 0.01 K/ps

To examine the effect of heating rate on structure evolution during the heating process, the structural evolution of the nanowire was observed at the initial (298 K) and final (698, 898, and 1098 K) for each rate as shown in Fig. 6. At the highest rate (1.0 K/ps), only a slight distortion is visible, with minimal atomic diffusion or rearrangement near the contact region. This indicates that rapid heating limits the time available for atomic reorganization, as kinetic energy increases too quickly for thermally activated processes such as diffusion to occur. At a moderate heating rate (0.1 K/ps), structural evolution becomes more evident, with noticeable rearrangements near the contact region. The lowest heating rate (0.01 K/ps) produces the most significant structural change. The interface is reconstructed, and the nanowire configuration is significantly disordered due to substantial atomic rearrangements and diffusion, enabled by extended heating time. In addition to the heating rate, the maximum heating temperature has a significant impact on structural evolution. Previous in situ TEM studies on metallic nanowires (e.g., Ag) have shown that atomic migration, neck formation, and fusion become pronounced above critical temperatures (~ 500–600 °C) [48]. Similarly, in this study, heating to 1098 K enhances atomic mobility and diffusion, leading to more substantial structural rearrangements and a fivefold twin-axis reconstruction.

The *\:MSD* and $\:{R}_{g}^{xx}$ graphs, along with the snapshots of atomic configurations presented in this section, highlight the critical roles of the heating rate and the final temperature in determining the crystalline structural response during sintering. Slower heating results in significant structural evolution, whereas rapid heating tends to “maintain” the original structural configuration within the same temperature range, due to insufficient time for diffusion. This observation on the heating rate effect may be helpful for designing sintered nanowire networks by carefully tailoring temperature and heating rate.

### Uniaxial tensile behaviors

#### Strain and heating rate effects

**Fig. 7:**
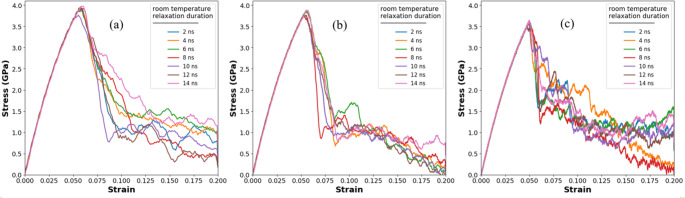
Tensile stress-strain ($\:{\sigma\:}_{zz}$ vs. $\:{\epsilon\:}_{zz}$) responses of sintered products at 698 K at a heating rate of 0.01 K/ps subjected to uniaxial tensile strain rate of (**a**) 1.0 × 10^− 3^, (**b**) 5.5 × 10^− 4^, and (**c**) 1.0 × 10^− 4^ ps^− 1^, after different room-temperature relaxation durations

To further understand the influence of sintered temperature and heating rate on mechanical properties, tensile testing was performed on the sintered cross-shaped models at three different strain rates along the *z*-direction. The differences in tensile performance of samples that underwent a similar sintering process from 698 K with a heating rate of 0.01 K/ps, but with different strain rates, are compared. Figure 7 illustrates how strain rate affects UTS, which can be considered equivalent to the yield strength reached at the end of the elastic regime. The result indicates that a yield strength of ~ 3.9 GPa, which is the highest of all the strengths, is obtained at the highest strain rate of 1.0 × 10^− 3^ ps^− 1^, while a yield strength of ~ 3.6 GPa, which is the lowest yield strength, is obtained at the lowest strain rate of 1.0 × 10^− 4^ ps^− 1^, which falls within the predicted range, consistent with experimental observation [35, 55]. This indicates that the yield strength increases with the increase in strain rate. Below this point, stress rises linearly as the strain increases up to about 5% in all cases. The linear stress–strain curves overlap with each other, showing that the strain rate has little effect on the elastic region. Consequently, Young’s modulus remains constant across different strain rates. Beyond the elastic limit, stress drops sharply as the yield point is passed, indicating the start of plastic deformation. The applied strain rate is strongly shown to affect stress-strain behavior during plasticity. At lower strain rates, the curve shows a clear zig-zag pattern, with repeated rises and falls in stress as the strain increases; however, the magnitudes of these fluctuations vary rather than remaining constant until fracture occurs near 20% strain. In contrast, at higher strain rates, the rise-and-fall patterns disappear, leaving only minor fluctuations due to strain hardening. Furthermore, the stress-strain curves shown in Fig. 7 were obtained using different relaxation durations, from 2 ns to 14 ns, at 298 K. These curves nearly overlap, with only slight variations in the peak stress, indicating that the relaxation time has a minimal effect on the UTS and deformation behavior. The consistent elastic and plastic responses across all cases in Fig. 7 indicate that the mechanical response is primarily governed by strain rate, instead of relaxation duration. A similar trend is also observed in other models sintered at 898 and 1098 K.

**Fig. 8:**
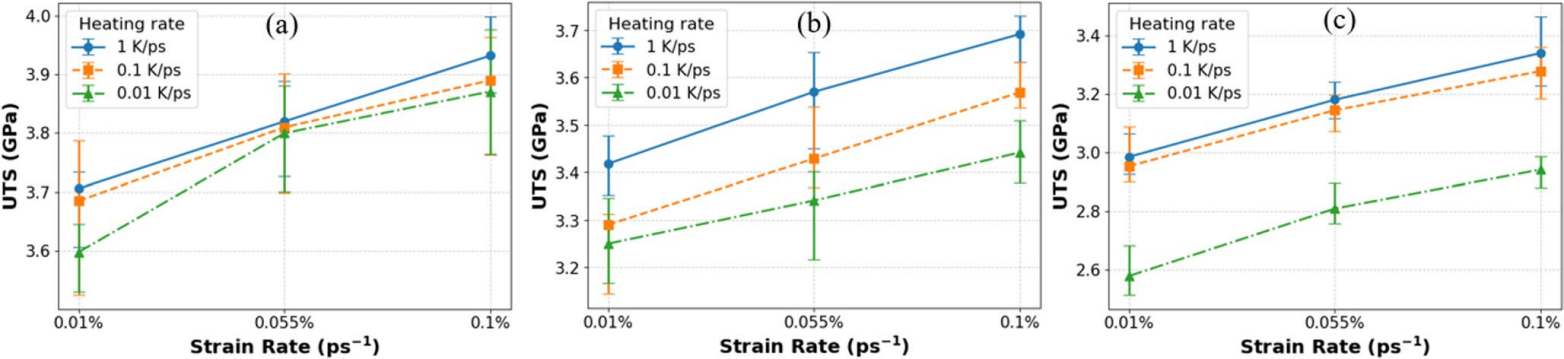
Averaged UTS vs. Strain rate of the sintered FTNWs obtained from sintered temperature of (**a**) 698, (**b**) 898, and (**c**) 1098 K, with different room-temperature relaxation durations

Figure 8 shows the variation in the averaged UTS across samples with different relaxation durations, under different combinations of strain rate, sintered temperature, and heating rate. UTS increases with increased strain rate; the highest rate produces the greatest strength, while the lowest rate gives the weakest. However, UTS decreases with increased sintered temperature. As shown, for the same heating and strain rates, the curves at 1098 K have the lowest values, followed by those at 898 K, while those at 698 K have the highest values. The error bars indicate the standard deviations of grouped values obtained under different relaxation conditions relative to their means; in all cases, most deviations are relatively small, suggesting good reproducibility of the simulated results. The heating rate shows only a relatively smaller influence on UTS at the same sintered temperature. As shown in each panel of Fig. 8, the datasets for 1.0, 0.1, and 0.01 K/ps are very close to each other (less than 0.5 GPa on average), with the 1.0 K/ps dataset producing marginally higher UTS values. At 1098 K (see Fig. 8(c)), the UTS values for higher heating of 1.0 and 0.1 K/ps are very close. However, a slight difference in UTS is observed when the sample is heated at a very low heating rate (0.01 K/ps) at this temperature. This occurs because, at the higher sintered temperature, there is a significant variation in the original internal twinned structure. Slow heating further provides sufficient time for atomic rearrangements, altering the twinned structure and ultimately reducing UTS. Overall, the results clearly indicate that both strain rate and sintered temperature are the primary factors controlling UTS, while the heating rate contributes in a second important way in influencing the strength of FTNWs.

Figure S1, in the supplementary information, shows sintered nanowires at 698 K and a heating rate of 1.0 K/ps under varied strain rates. The results were obtained after a fixed 1 ns of room-temperature relaxation, during which different atomic velocity distributions were generated with distinct random seeds within the same ensemble. Similarly, Fig. S2 summarizes the variation in the average UTS over samples of velocity distributions. They show a similar trend to Figs. 7 and 8, where UTS increases with increased strain rate. For a given strain rate, the results indicate that the UTS remains nearly identical, suggesting that strength depends primarily on the applied loading rate rather than on the initial atomic velocity distributions used for relaxation. However, different initial velocity distributions can trigger differences in detailed post-yield behavior. As shown in Figure S1, necking and eventual fractures do not consistently occur at the same location, even under identical thermal and mechanical conditions. This suggests that slight variations in the initial atomic configuration can significantly impact the detailed plastic deformation and strain-hardening behavior even when the same thermal and loading conditions are applied. This is also true for models sintered at 898 and 1098 K, as a similar trend is observed with the same relaxation time duration but different velocity distributions.

#### Sintered temperature effect

**Fig. 9:**
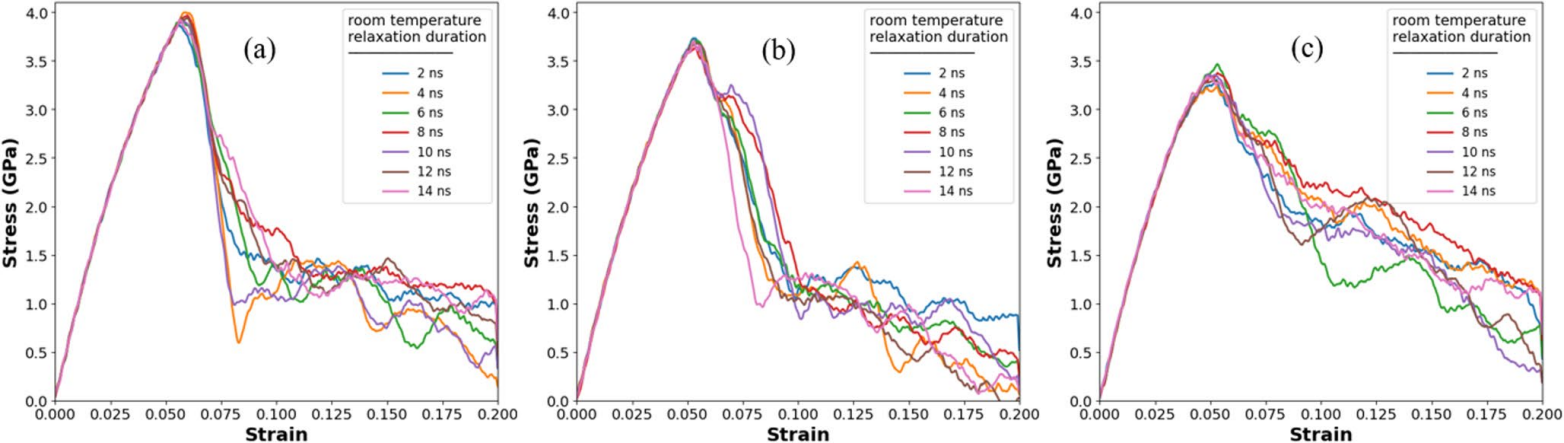
Tensile stress-strain ($\:{\sigma\:}_{zz}$ vs. $\:{\epsilon\:}_{zz}$) curves for FTNWs sintered at (**a**) 698, (**b**) 898, (**c**) 1098 K, and at a heating rate of 1.0 K/ps, subjected to uniaxial tensile loading at 1.0 × 10^− 3^ ps^− 1^, after different room-temperature relaxation durations

**Fig. 10:**
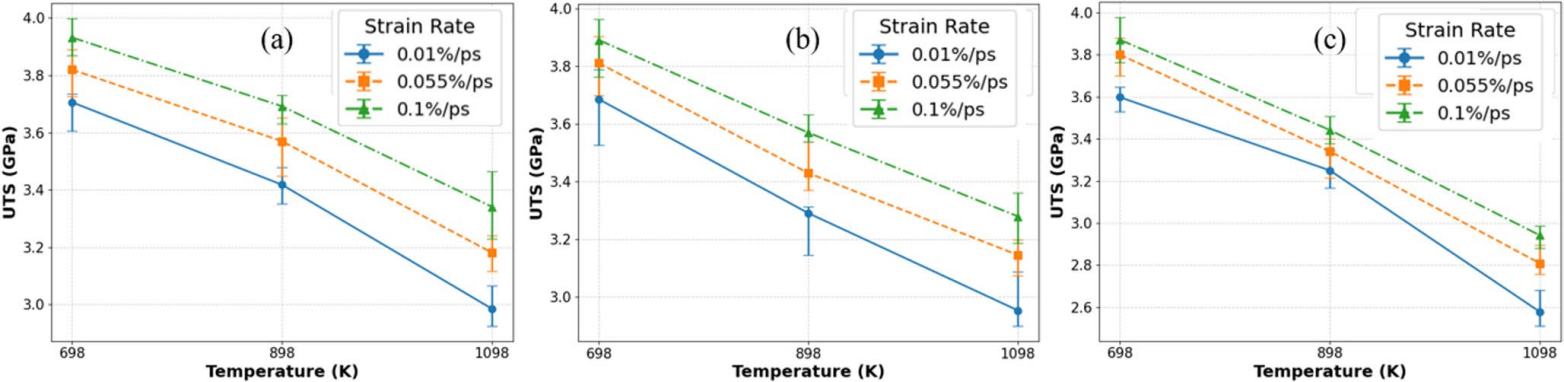
Averaged UTS vs. Temperature of the sintered FTNWs obtained from heating rate of (**a**) 1.0, (**b**) 0.1, and (b) 0.01 K/ps, after different room-temperature relaxation durations

To further study the sintered temperature effect, Figs. 9 and S3 are plotted to show the tensile stress-strain responses of sintered nanowires at a strain rate of 1.0 × 10^− 3^ ps^− 1^ and sintered temperatures of 698, 898, and 1098 K with a heating rate of 1.0 K/ps under different relaxation durations and velocity distributions, respectively. In addition, Figs. 10 and S4 are shown to summarize the corresponding averaged UTS obtained from different combinations of temperature, strain rate, and heating rate. The sample sintered at 698 K shows the highest tensile strength across all strain rates. This is because at a lower sintered temperature, the diffusion of atoms is lower, which helps preserve the internal TBs. The fivefold twinned model tends to retain all its stress along the central axis, and the central axis undergoes less structural change (see Fig. 6), which contributes to its higher tensile strength. In contrast, the higher-temperature sintered sample allows for greater atomic mobility, which facilitates increased dislocation activity near TBs and neck connections and thus triggers greater changes to the original fivefold pattern, leading to a reduction in structural strength. Quantitatively, raising the sintered temperature from 698 to 1098 K results in a roughly 20–30% reduction in strength. Despite the reduction in strength at the higher sintered temperature, the sample shows a smoother stress decrease with more gradual post-yield softening, suggesting improved ductility mediated by more SF and twinning activities. However, at low sintered temperatures with increased strength, it shows a sharp drop in stress after reaching the yield point, indicating more brittleness before fracture. Overall, the results demonstrate that the sintered temperature is the dominant factor governing the strength and ductility of sintered FTNW structures: low-temperature sintering preserves the original fivefold-axis strength but limits ductility. In contrast, high-temperature sintering lowers strength but enhances ductility. It should be noted that such sintered temperature effects on FTNW models are opposite to those of sintered chain products of single-crystal nanoparticles [22], which are governed by a different deformation mechanism due to the absence of FDTs.

In summary, the comparative analysis of Figs. 9 and 10 and S3-S4 shows that varying the room-temperature relaxation duration or applying different random seeds to initiate atomic velocity distributions does not produce any significant changes in the UTS results. However, these two factors are helpful in generating more independent samples under identical sintering conditions, which yield more solid results with better sampling. The observation of our present study suggests that using a relatively low sintered temperature (698 K) with a faster heating rate is optimal in the sintering design of an FTNW network for better mechanical strength. The results clearly show that the dominant factor controlling the strength and deformation response is the sintered temperature, which governs atomic diffusion and the stability of the fivefold twinned axis.

#### Tensile stress-strain response and atomic-level deformation mechanisms

**Fig. 11:**
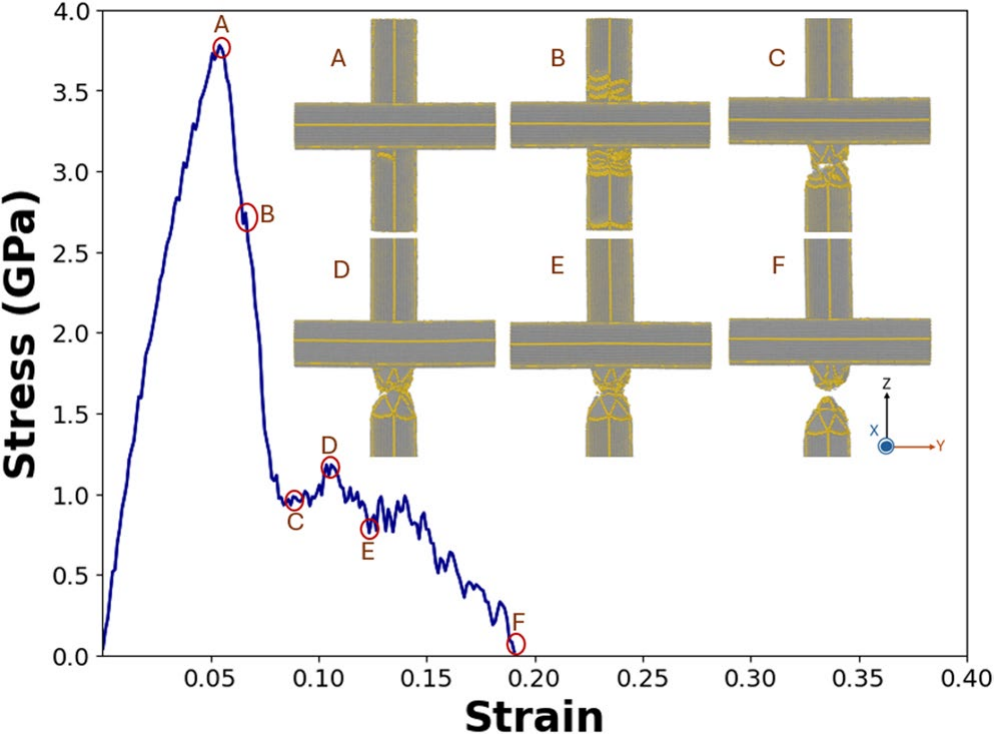
Tensile stress-strain ($\:{\sigma\:}_{zz}$ vs. $\:{\epsilon\:}_{zz}$) response of the cross-shaped nanowires sintered at 698 K at a heating rate of 0.01 K/ps and strain rate of 5.5 × 10^− 4^ ps^− 1^, along with the atomic configuration snapshots showing the formation of SFs and FDTs. Points A to F represent various stages of nanowire deformation from peak stress to necking and finally fracture

A typical stress-strain curve is shown in Fig. 11, plotted for the cross-shaped model sintered at a temperature of 698 K, heated at 0.01 K/ps, and tested at a strain rate of 5.5 × 10^− 4^ ps^− 1^. This figure also illustrates the process of plastic deformation, necking, and fracture. It can be seen that the tensile stress increases with the increase of strain from 0% up to ~ 5%, where it reaches a peak of 3.76 GPa within the elastic regime (see point A). As it reaches this point, some atoms start to slip along $\left\{111\right\}$ planes, forming an SF led by the motion of dislocation cores. The initial formation of the SF begins on the lower side of the connection region, as shown in Fig. 11 (see point A). This SF originated from the free surface of the twin domain, due to that the relatively high free-surface energy makes it easier to overcome the energy barrier to initiate such type of planar defects. Its further movement is restricted by the two pre-existing TBs that serve as the boundaries of the subdomain (i.e., nano-grain). Then, multiple SFs and dislocations are left along the wire length, as the strain continually increases. The decrease in stress starts once the SFs start to form and expand along the nanowire, indicating the onset of plastic deformation. This SF plays a significant role because it seems to trigger other adjacent SFs, and later they are connected and extended across all five subdomains, and finally, the nanowire experiences necking and fractures in the same region (see points E and F). Back at point B, as the stress starts to drop, other SFs start to originate from the upper side as well as the back of the connection region in the vertical nanowire. These SFs that are formed all initiate from the free surface of the twin domains. At point B, the fraction of HCP atoms increases rapidly from 13,173 to nearly 18,617 as the strain reaches approximately 6.5%. The increase in HCP atoms is directly related to the formation of multiple SFs, corresponding to the stress deduction, as evidenced by the drop from the peak value to 2.75 GPa at point B. The formation of these HCP atoms, which increases defect density, is significantly dependent on the strain rate, as shown in Fig. S5. At higher strain rates (1.0 × 10^− 3^ ps^− 1^), dislocation nucleation is rapid, and SFs (HCP regions) accumulate, resulting in higher peak defect densities. This is because at high strain rates, there is not enough time for partial dislocations to rearrange and annihilate SFs, leading to an increased HCP fraction.

At point C, the fraction of HCP atoms starts to decrease to 14,651, corresponding to approximately 10.4% of the total atoms, and then remains almost constant. Correspondingly, in the snapshot of the atomic configuration, the annihilation of SFs can be observed on the upper side of the connection. Additionally, in the lower region of the deformed nanowire, new TBs start to form from the previous intrinsic SF region, which is mediated by the motion of partial dislocation cores and the formation of intermediate extrinsic SFs, and these extrinsic SFs later develop into networks of TBs that form the secondary FDTs within each pre-existing twin subdomain. Interestingly, the necking appears in the same region, where several triangular loop structures can be observed and become the facets of the tetrahedral structure, as shown in the snapshots at points D and E. A similar local deformation twinning process in the necking zone was previously observed in copper nanowires under a combined pre-torsion and tension loading condition [36]. As the strain increases further, the nanowire becomes progressively thinner, breaking apart at a strain of approximately 19% (see point F). The nanowire, after fracture, divides into two parts, and each of them forms a stable quasi-icosahedral structure. Such a structure formed at the necking region is in good agreement with a previous study, which showed that hemisphere-like quasi-icosahedral caps form in low-SF-energy metals such as Cu, Ag, and Au during tensile deformation [37]. This quasi-icosahedral structure remains stable once it is formed, even after the nanowire breaks into two parts.

### Formation of FDTs and quasi-icosahedral structure

**Fig. 12:**
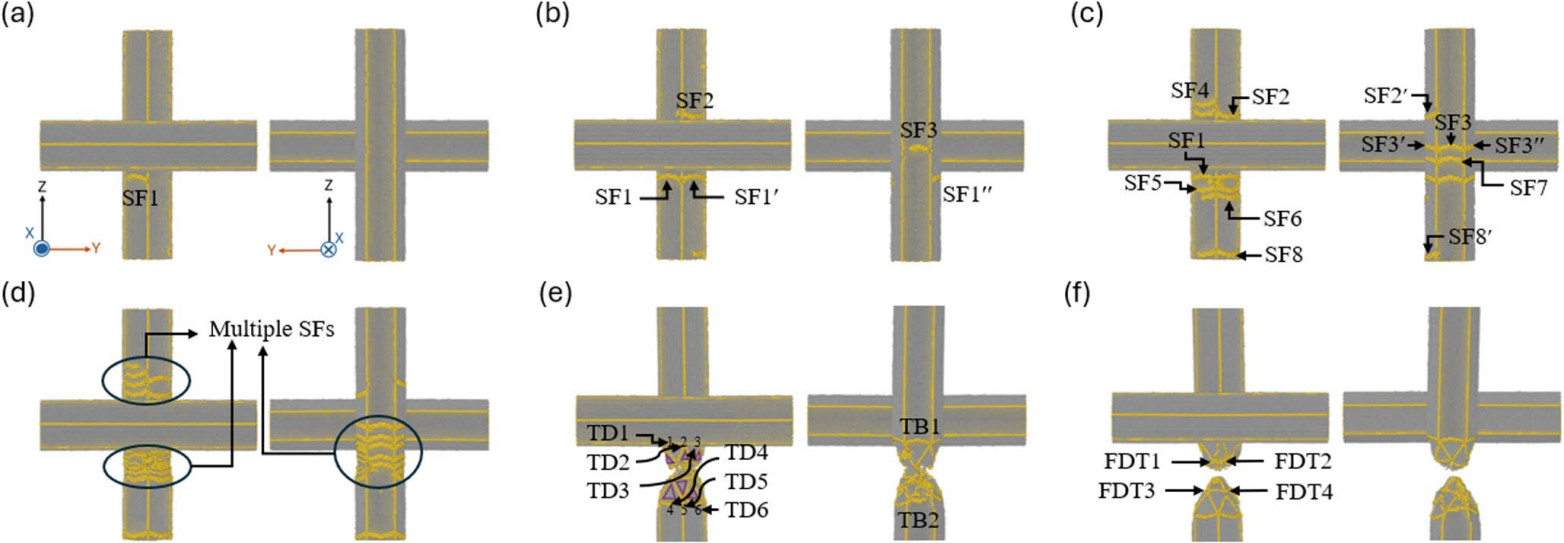
Snapshots captured at the strain of (**a**) 5.62%, (**b**) 5.83%, (**c**) 6.09%, (**d**) 6.73%, (**e**) 13.81%, and (**f**) 20%, showing the formation process of a quasi-icosahedral structure in cross-shaped FTNWs under a tensile strain rate of 5.5 × 10^− 4^ ps^− 1^. The cross-shaped model was obtained from sintering at 698 K at a heating rate of 0.01 K/ps

Figure 12 illustrates the detailed formation process of the quasi-icosahedral structure in cross-shaped nanowires, stretched at a strain rate of 5.5 × 10^− 4^ ps^− 1^. SFs, labeled by SF1 and SF2, are generated by the movement of $1/6\langle112\rangle$ Shockley partial dislocation cores of the $\left\{111\right\}$ planes. Initially, only SF1 is visible at a strain of 5.62%, as shown in Fig. 12(a); later, SF2 and SF3 become visible at a strain of 5.83%. All these SFs originate in a similar way, as they originate from the surface edges of the fivefold twin domains and later facilitate the formation of other secondary SFs; SF1 forms on the lower side of the vertical nanowire, SF2 on the upper side of the vertical nanowire, and SF3 in the connection region, located at the backside of the same nanowire, as viewed along the *x*-direction in Fig. 12(b). As discussed above, SF1 and SF2 propagate along the $\left\{111\right\}$ planes and later trigger several secondary SFs in the adjacent two subdomains (see Fig. 12(c)). Similarly, SF3 appears to facilitate the formation of secondary SFs (SF3′ and SF3″) in both adjacent subdomains, as they are initiated on the original TBs after SF3 reaches these boundaries, as illustrated in Fig. 12(c). Additionally, at the bottom of the vertical nanowire, a new SF8 originates; it forms in a similar way to other SFs and later connects with other secondary SFs in the other four twin domains, forming a complete loop as SF1 does. These SFs (SF1 - SF8) form through the same mechanism: each is initiated from the free surface of one twin domain, via the nucleation of dislocation cores and gliding along $\left\{111\right\}$ planes, and then is stopped by the pre-existing TBs on both sides. Upon reaching the subdomain boundaries, they tend to facilitate new dislocation cores nucleated on the original TBs, which form the secondary SFs (e.g., SF1″, SF2′, SF3′, SF3″, and SF8′). They then tend to extend and form networks, wrapping around the central fivefold axis, but only SF1 and SF8 succeed in doing so.

At a strain of 6.73%, multiple SFs accumulate, increasing the defect density within the nanowire, as depicted in the ellipse frames in Fig. 12(d). The density of these multiple SFs formed in distinct regions of the nanowire is different. More intense distributed SFs are found on the lower side of the nanowire and the connection area, rather than on the upper side of the connection. In addition, SF8, which was previously formed at the bottom of the vertical nanowire, along with its adjacent second SFs, later annihilates after the reorientation to the original FCC structures in these twinned subdomains, as shown in Fig. 12(e). Similarly, annihilation was also observed for SF2 and SF4. At a strain of approximately 13.81% in Fig. 12(e), a quasi-icosahedral structure develops during the necking as the strain increases. The SFs on the lower side of the sintered junction are gradually separated by layers of FCC atoms and begin to form new TBs. Specifically, two sets of TBs on both sides of the necking, TB1 and TB2, as labeled in the second panel of Fig. 12(e), start to form several triangular loops that play a significant role in the formation of the quasi-icosahedral structure. The loops, marked by triangles in the first panel of Fig. 12(e), represent the facets of the newly formed tetrahedral subunits, designated as TD1-TD6. These subunits combine with the tri-junction and form the secondary FDTs (FDT1, FDT2, FDT3, and FDT4), as illustrated in Fig. 12(f). A similar formation process of the quasi-icosahedral cap is found on the opposite side of the necking.

**Fig. 13:**
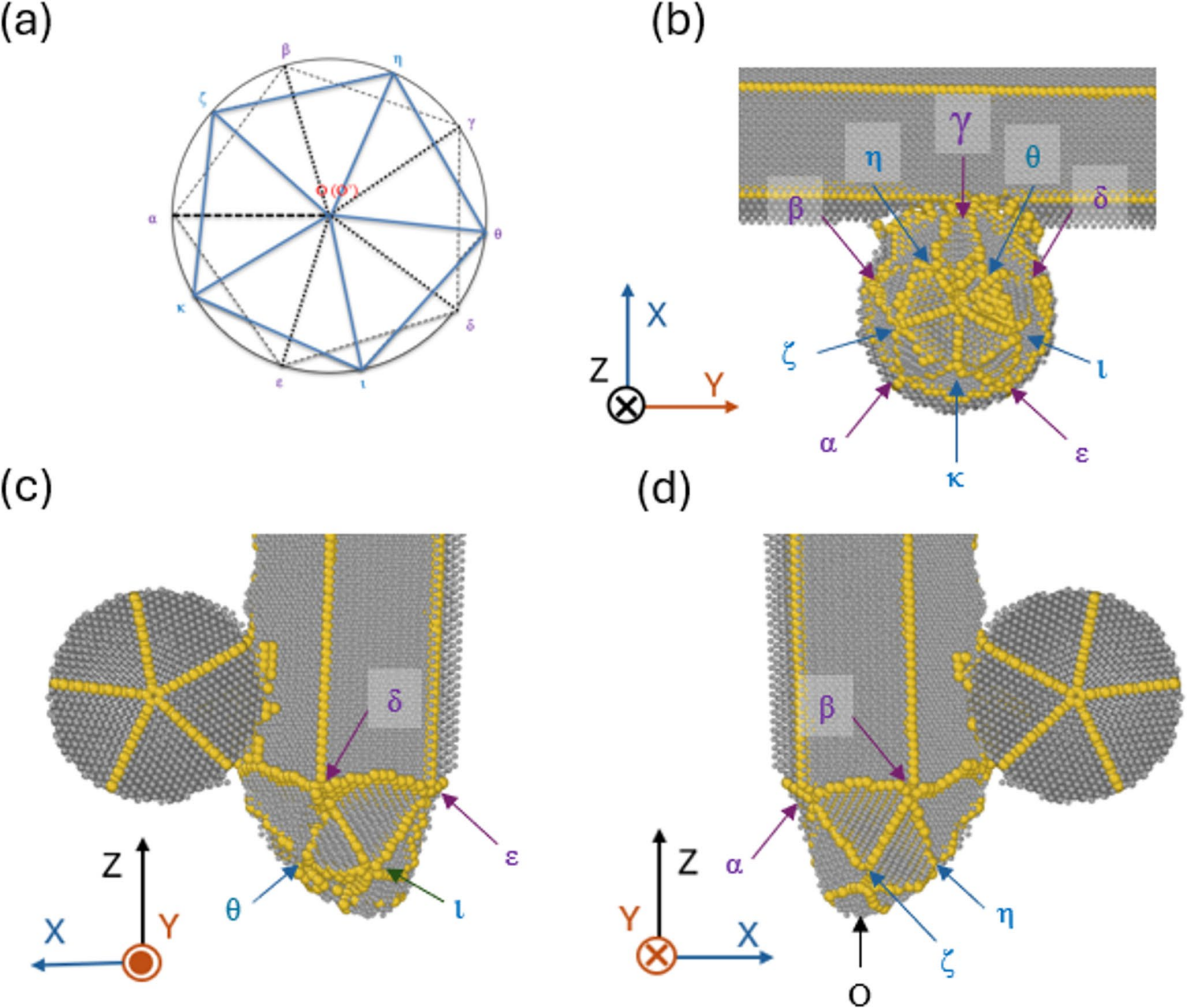
(**a**) The sketch of an ideal icosahedron, and sliced atomic configurations in (**b**) top view, (**c**) right-side view, and (**d**) left-side view, showing the shape and composition of a quasi-icosahedral structure formed in cross-shaped FTNWs after fracture

Figure 13(a) shows a sketch of an ideal icosahedron with its 12 vertices orthogonally projected on a 2-D plane. The letter labels represent the loci of fivefold symmetry centers, and the 30 edges correspond to both intrinsic and deformation TBs. The quasi-icosahedral structures observed on the necking surface of the nanowires are shown in other panels of Fig. 13. In the fractured region, a quasi-icosahedral structure comprises a twisted original fivefold twinned axis combined with ten secondary FDTs, creating a complex but “perfect” arrangement of five preexisting and fifteen tetrahedral subunits joined adjacently to each other. Unlike an ideal icosahedron with 20 identical tetrahedra, these structures contain only 15 tetrahedral FCC subunits, with the remaining five subunits being the original quasi-triangular prisms that enclose a wire cross-section. The formation process involves successive twinning mechanisms initiated at the necking region during the short duration of plastic deformation before fracture. As discussed above, the multiple SFs are blocked by TBs, leading to micro-twins that evolve into single TBs and eventually form tetrahedral subunits through the formation of triangular loops [37]. This unique multiple-twinned structure divides the fracture surface into fifteen small triangular facets, creating hemispherical caps at the fracture point. Thus, the quasi-icosahedral structures formed in FTNWs possess only 11 fivefold centers (designated as *O* and *α-κ*) that develop in the fractured region, accompanied by a long intrinsic fivefold axis (designated as *O’*). The final FDTs, denoted as *O*, maintain coaxial alignment with the wire’s center *O’*. This arrangement of twin centers may serve to minimize local strain energy at the fracture interface, promoting structural stabilization through angular redistribution of stress. The centers of the first group of FDTs (*α-ε*) are positioned on a single plane that intersects perpendicularly with the nanowire axis (see panel (b)). These five centers are situated adjacent to the undeformed portion of the nanowire and share the preexisting TBs with the original fivefold axis of the wire. The second group of FDT centers (*ζ-κ*) lies in a different plane, closer to the breakpoint, as can be seen in panels (c) and (d). Each of these five centers contributes one TB of the top central FDT. This geometric configuration creates a complex network of FDTs that stabilizes the quasi-icosahedral structure following fracture [37]. It should be noted that the formation of such a quasi-icosahedral structure is also observed in tension tests of this study with the other strain rates, including 1.0 × 10^− 4^ ps^− 1^ and 1.0 × 10^− 3^ ps^− 1^, respectively.

### Mechanical behavior comparison of sintered SCNWs and FTNWs under tension

**Fig. 14:**
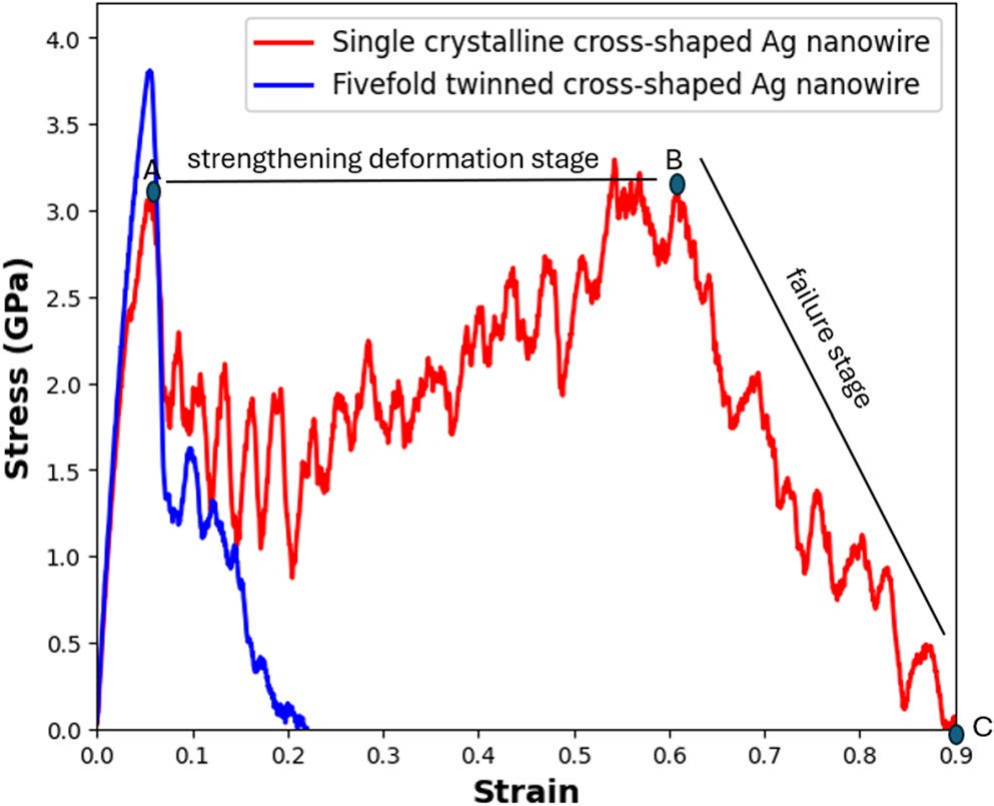
Stress-strain ($\:{\sigma\:}_{zz}$ vs. $\:{\epsilon\:}_{zz}$) curves of cross-shaped SCNW and FTNW, sintered at a temperature of 698 K at a heating rate of 1.0 K/ps under a uniaxial tensile strain rate of 5.5 × 10^− 4^ ps^− 1^

To further demonstrate the unique deformation behavior of sintered FTNWs $\langle110\rangle$ SCNWs with similar dimensions were constructed and tested under tension. The mechanical response of SCNWs and FTNWs was compared under identical tensile conditions: sintered at 698 K with a heating rate of 1.0 K/ps, followed by uniaxial loading along the *z*-direction at a strain rate of 5.5 × 10^− 4^ ps^− 1^. Figure 14 indicates that, within the first stage of the elastic regime, the two nanowires display nearly identical behavior and comparable elastic moduli. However, the yield strength of the fivefold twinned model is 3.78 GPa, approximately 22% higher than the 3.10 GPa of the single crystal, due to the fact that TBs with a high pre-stress state require higher strain energy for further crystalline changes. This increase is a considerably high strength increase in the nanowire systems.

Beyond the elastic region, the stress drops sharply for both models, with a more pronounced decline for the FTNW. Its maximum strain before fracture (around 0.2) is about 4.5 times lower than that of the SCNW, indicating reduced ductility in FTNWs. This limited ductility is attributed to the pre-existing fivefold TBs, which act as barriers to the emission and propagation of partial dislocations. In contrast, the single crystal shows two distinct post-yield stages: a strengthening/strain-hardening stage (A-B) and a failure stage (B-C), as shown in Fig. 14. In the first stage, from point A to B, when the strain value exceeds 0.05, the stress value decreases due to the nucleation and movement of dislocation cores, marking the beginning of plastic deformation. As the strain increases, the nanowire starts to transit to another crystal orientation along the longitudinal directions mediated by the movement of stacking faults and the twinning process, reaching a stress value even above the previous yield stress, resulting in a higher UTS at point B. The UTS of a single crystal is crucial, as it reflects the maximum stress that it can withstand before fracturing. It shows that SCNW exhibits good ductility and displays higher strength than its elastic limit after plastic deformation, as its ultimate strength (point B) exceeds its yield strength (point A). This phenomenon is unique for SCNWs and is not observed in FTNWs. After point B, the material stress drops sharply, and the nanowire fractures at point C when the strain value reaches 0.9.

The two nanowire types also differ in deformation mechanisms. FTNWs exhibit a unique ability to form quasi-icosahedral structures via successive twinning processes localized within the necking region. These complex configurations consist of a twisted original fivefold twinned axis accompanied by secondary FDTs. However, SCNWs subjected to identical tension do not form quasi-icosahedral structures. The absence of preexisting TBs in the single-crystalline configurations, with no internal concentrated stress state, is the main reason that fundamentally limits their capacity to nucleate and form the complex multi-twinned arrangements that are commonly observed in the fivefold twinned models. Instead, SCNWs exhibit dislocation of the planes during plastic deformation, which does not facilitate geometric reorganization that could lead to the formation of quasi-icosahedral structures.

## Conclusion

In this work, a comprehensive MD study was conducted to examine how sintering conditions affect the mechanical behavior of cross-shaped FTNWs under various thermal and mechanical conditions. Precisely constructed FTNWs were sintered at 698, 898, and 1098 K with heating rates of 1.0, 0.1, and 0.01 K/ps, followed by high-temperature relaxation, controlled cooling, and room-temperature relaxation for different durations of time and applying random seeds under the same ensemble to generate statistically independent initial velocity distributions. Variations in relaxation duration and velocity initialization produced negligible changes in UTS, although post-yield deformation behaviors were affected. Sintering temperature emerged as a dominant factor: lower temperatures preserved fivefold TBs and promoted higher strength, whereas elevated temperatures enhanced diffusion, degraded TBs, and reduced tensile performance. Under uniaxial tension, FTNWs show pronounced strain-rate sensitivity, with yield strength increasing from 3.4 GPa at 1.0 × 10^− 4^ ps^− 1^ to 3.98 GPa at 1.0 × 10^− 3^ ps^− 1^ (around 117.06% increase), while the elastic modulus remains constant, confirming that strain rate primarily governs strength and plastic deformation mechanisms. Low strain rates produced zigzag stress-strain responses, indicating sequential dislocation nucleation and propagation, whereas higher rates produced smoother, continuous flow behavior. Plastic deformation initiated with Shockley partial dislocations and SF formation at around 4.6% strain, followed by SFs-to-TBs transformation around 9.95% strain and the development of tetrahedral subunits that assembled into quasi-icosahedral structures at fracture. Comparative analysis with SCNWs for the same condition shows that FTNWs achieved around 22% higher yield strength (3.78 GPa vs. 3.10 GPa) due to TB-mediated dislocation blocking, but with a substantial ductility reduction, fracturing at around 20% strain compared with around 90% for single-crystalline structures, representing a 4.5 times reduction in ductility. FTNWs show a sharp drop in stress immediately after yielding, with limited strain hardening before failure. In contrast, the single-crystalline one shows distinct hardening behavior after a slight stress drop beyond the yield point. During the failure process, FTNWs can form the quasi-icosahedral structure through the secondary twinning process, whereas SCNWs deform through conventional dislocation mechanics without any complex structure transformation before failure.

This research offers critical insights into how sintering history impacts the yield strength, deformation mechanisms, and failure patterns of FTNWs, and is expected to provide theoretical guidance for potentially optimizing the durability of nanoelectromechanical systems subjected to combined thermal and mechanical stresses.

## Supplementary Information

Below is the link to the electronic supplementary material.

Supplementary Material 1

## Data Availability

Yes, I used or generated research data in this study. The data supporting the findings of this study can be provided upon request. Please email the corresponding author at jiang@olemiss.edu for any data needed.
