# Supplementary material for: Mechanical behavior of sintered cross-shaped fivefold twinned Ag nanowires: Insights from a molecular dynamics study
Source: Appl Phys A Mater Sci Process. 2026 Jun 10;132(7):602. doi: 10.1007/s00339-026-09778-7 (PMC13253788; doi:10.1007/s00339-026-09778-7)
Supplement: Supplementary file 1 — Supplementary Material 1 [file 339_2026_9778_MOESM1_ESM.docx]

**Supplementary Information**

**Mechanical behavior of sintered cross-shaped fivefold twinned Ag nanowires: Insights from a molecular dynamics study**

Prabesh Ojha^1^, Huadian Zhang^1^, Manoj K. Shukla^2^, Michael R Fiske^3^, Jennifer E Edmunson^4^, and Shan Jiang^1, *^

^1^ Department of Mechanical Engineering, University of Mississippi, University, MS, 38677, USA

^2^ Environmental Laboratory, U.S. Army Engineer Research and Development Center, Vicksburg, MS 39180, USA

^3^ Amentum, Space Exploration Division, NASA/Marshall Space Flight Center, Huntsville, AL 35812, USA

^4^ ST23/Space Technology Development Branch, NASA/Marshall Space Flight Center, Huntsville, AL 35812, USA

* Address correspondence to E-mail: [jiang@olemiss.edu](mailto:jiang@olemiss.edu)


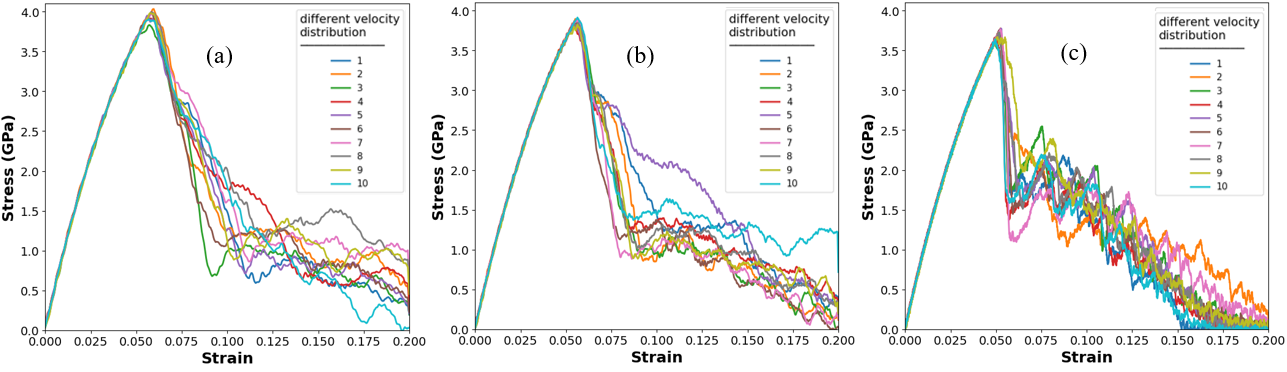


Fig. S1 Tensile stress-strain ($\sigma_{zz}$ *vs.* $\varepsilon_{zz}$) responses of sintered products at 698 K with a heating rate of 0.01 K/ps subjected to uniaxial tensile loading at a strain rate of (a) 1.0×10^-3^, (b) 5.5×10^-4^, and (c) 1.0×10^-4^ ps^-1^, after room-temperature relaxation for 1 ns with different random seeds to generate velocity distributions.


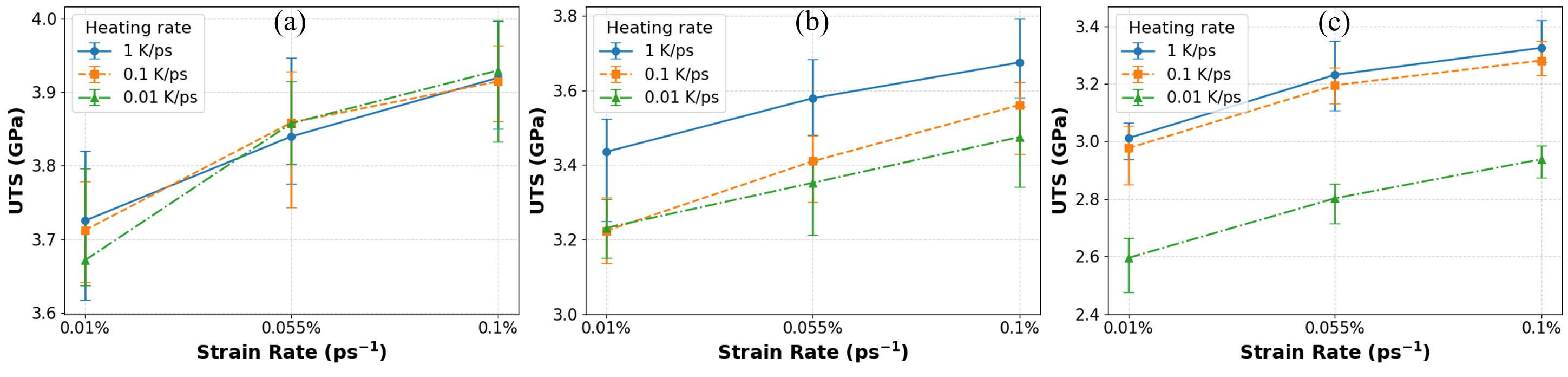


Fig. S2 Averaged UTS *vs.* strain rate of the sintered FTNWs obtained at sintered temperature of (a) 698, (b) 898, and (c) 1098 K, after room-temperature relaxation for 1 ns, with different random seeds to generate velocity distributions.


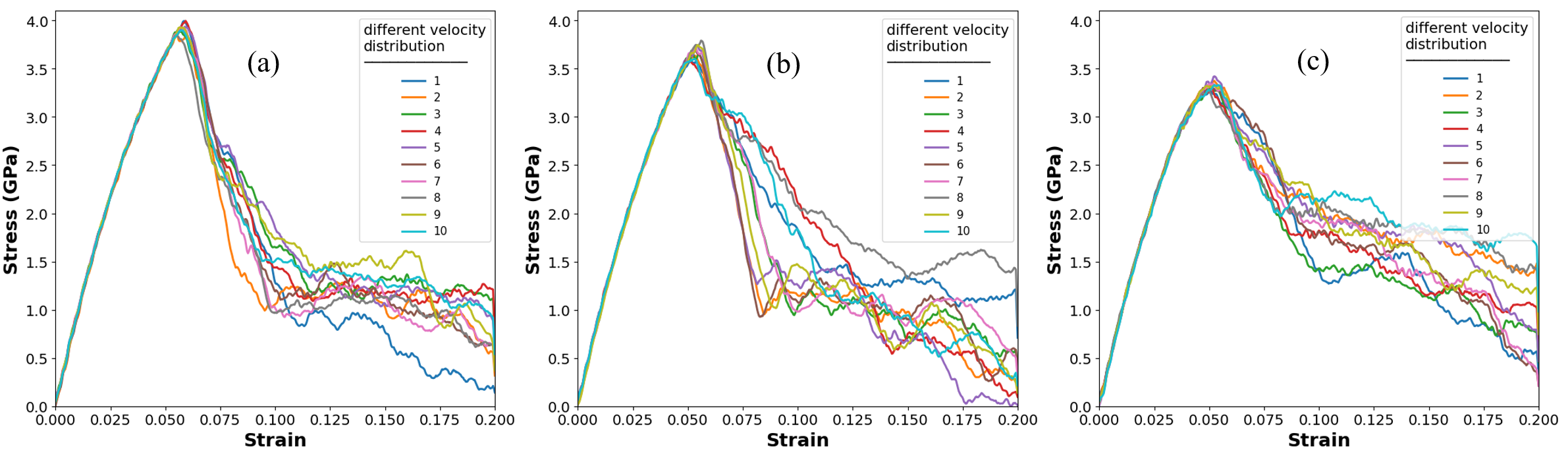


Fig. S3 Tensile stress-strain ($\sigma_{zz}$ *vs.* $\varepsilon_{zz}$) curves for FTNWs sintered at (a) 698, (b) 898, (c) 1098 K, and at a heating rate of 1 K/ps, subjected to uniaxial tensile loading at a strain rate of 1.0×10^-3^ ps^-1^ after room-temperature relaxation for 1 ns, with different random seeds to generate velocity distributions.


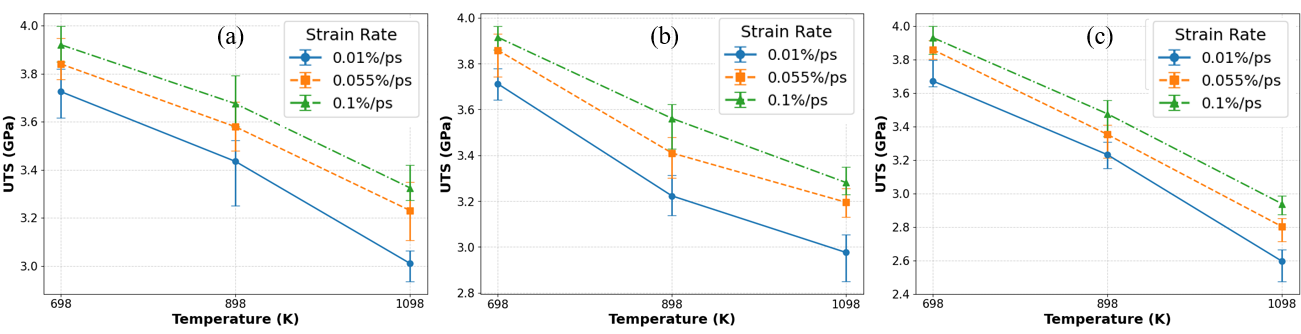


Fig. S4 Averaged UTS *vs.* temperature of the sintered FTNWs obtained at a heating rate of (a) 1.0, (b) 0.1, and (c) 0.01 K/ps after room-temperature relaxation for 1 ns with different random seeds to generate velocity distributions.


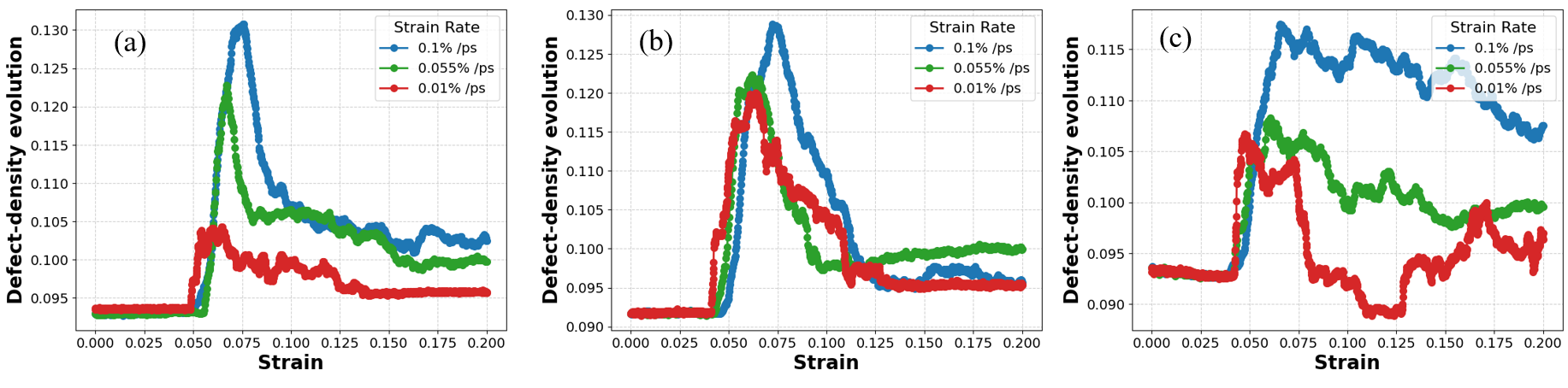


Fig. S5 Defect density evolution (HCP fraction) vs. strain at a heating rate of 1.0 K/ps and sintered temperature at (a) 698, (b) 898, (c) 1098 K.
